# Colorimetric Quantification of Dopamine as a Bioactive Compound in Banana Peel Extracts: A Sustainable Approach to Food Waste Valorization

**DOI:** 10.1021/acsomega.6c03329

**Published:** 2026-06-15

**Authors:** Doretta Cuffaro, Enrico Crispino, Elisa Nuti, Vincenzo Calderone, Pasquale Palladino, Maria Minunni

**Affiliations:** † Department of Pharmacy, 9310 University of Pisa, Via Bonanno 6, Pisa 56126, Italy; ‡ Department of Chemistry “Ugo Schiff”, University of Florence, Via della Lastruccia, 3, Sesto Fiorentino (FI) 50019, Italy

## Abstract

Dopamine (DA) is a bioactive catecholamine highly concentrated in banana peel (BPEEL), a common agri-food waste. In this study, a rapid and sustainable colorimetric method for DA quantification in BPEEL extracts was developed, based on the formation of a purple-blue melanochrome (MN) chromophore in a Mg^2+^/NH_4_^+^ buffer (150 mM each, pH 9.4) in DMSO/H_2_O (1:1 v/v), as confirmed by UV–vis analysis. The strong absorbance at 595 nm was monitored by standard microplate spectrophotometry. The assay was applied to quantify DA equivalent (DAE) in BPEEL extracts, yielding 5000 ± 200 μg DAE/g dry matrix, consistent with UPLC-DAD analysis (5700 ± 300 μg DAE/g) used as the validation technique, with excellent linearity and reproducibility (*R* ^2^ = 0.9974, _av_RSD % = 1.94%). The colorimetric method’s detection and quantification limits were 1.8 ± 0.4 μg/mL and 5.4 ± 1.1 μg/mL, respectively, suitable for the expected DA levels in banana waste. Operating at the microliter scale and requiring minimal sample preparation, the proposed approach is fast, low-cost, and matrix-compatible, offering a simple and cost-effective analytical tool suitable for bioactive compound screening in food waste matrices.

## Graphical abstract

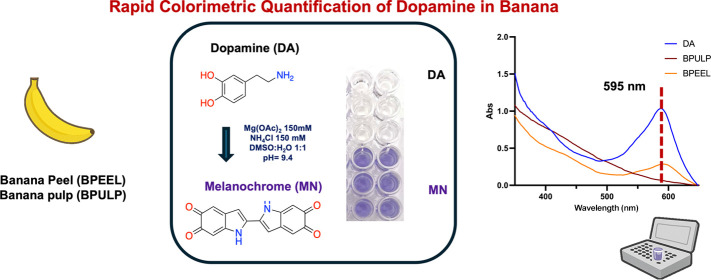

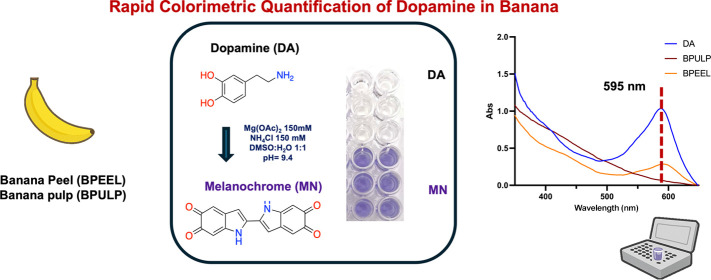

## 1 Introduction

Dopamine (DA) is a biologically relevant catecholamine that plays a fundamental role in human physiology.^1^ As a key neurotransmitter, it regulates motivation, reward, learning, and motor control by modulating synaptic transmission within specific neural circuits. Under physiological conditions, finely tuned dopaminergic signaling shapes behavior, cognition, and movement. Conversely, dysregulation of dopamine pathways has been strongly associated with a wide range of neurological and psychiatric disorders.^1,2^ In pharmacological settings, DA is used as a therapeutic agent due to its dose-dependent effects on dopaminergic, adrenergic, and cardiovascular systems, and it is commonly administered in the management of shock, heart failure, and severe hypotension to improve cardiac output and organ perfusion.^1^ Beyond its biomedical relevance, DA has attracted increasing interest in materials science and analytical chemistry due to its oxidative polymerization into polyDA and melanochrome (MN)-like structures. These reactions have been exploited in surface coatings, molecularly imprinted polymers, sensing platforms, and bioanalytical applications.^3–6^ In parallel, DA also plays important physiological roles in plants, where it is involved in stress responses, pigmentation, antioxidant defense mechanisms, and other biochemical processes relevant to food chemistry.^7^ In particular, reported DA levels in plant-based foods varies across species, resulting high in banana peel (∼700 μg/g dry weight), moderate in banana pulp (∼8 μg/g fresh weight) and avocado (∼4–5 μg/g fresh weigh), and low in oranges, tomatoes, spinach, peas, beans, and other fruits and vegetables (<1 μg/g).^7–9^ Given the large-scale global production of bananas and the consequent generation of peel as an agri-food waste, banana peel represents an underexploited yet highly concentrated natural source of DA and related catechol compounds. In this context, the reliable quantification of DA in banana peel extracts is essential not only for nutritional and biochemical characterization but also for supporting food waste valorization strategies aligned with circular economy principles.

Over the last five years, DA determination in plant-derived matrices and food-related samples has been predominantly addressed using chromatographic techniques, mainly HPLC or UPLC coupled to electrochemical or UV–vis detection.^9–11^ While these approaches provide high sensitivity and selectivity, they rely on expensive instrumentation, trained personnel, time-consuming sample preparation, and relatively high solvent consumption, which limit their applicability for rapid or onsite analyses.

Recently, our research group has focused on developing sensitive and sustainable analytical approaches for the detection of bioactive molecules in food^12,13^ and food waste matrices,^14^ aiming to enhance the valorization of these often-overlooked resources and promote circular economy strategies. In particular, considering that the Food and Agriculture Organization of United Nations (FAO) estimated a worldwide production of banana to be about 140 million tons/year,^15^ the development of a simple analytical approach for DA detection and quantification suitable for potential onsite use represents a relevant challenge in food analysis and bioanalysis. In line with our previous work,^16^ we developed an optimized method for DA identification in the food matrix, using banana samples and in particular banana peel and pulp (BPEEL and BPULP) as a case study.

This assay, where DA is quantified as dopamine equivalents (DAE), is based on the peculiar oxidative conversion of DA to melanochrome (MN) under mild alkaline conditions in DMSO/buffer 1:1 (v/v), a stable chromophore that produces a visible purple/blue color and can be quantitatively detected at 595 nm.^16^ Following optimization of extraction conditions, an ultraperformance liquid chromatography method (UPLC-DAD) was developed to enable quantitative analysis of DA in both matrices and to validate the colorimetric results. Moreover, the kinetics of MN formation were evaluated, describing the optimal reaction time for the subsequent analyses, balancing the signal development with the analytical reproducibility of assay response across samples. Results from the colorimetric and chromatographic approaches were compared, and the key analytical performance parameters of both methods were evaluated.

Although MN formation from DA has been previously exploited in pharmaceutical and biological contexts, this study represents the first systematic application of this chemistry to food waste analysis, validated through a matrix-matched UPLC-DAD reference method. The proposed approach enables the selective conversion of DA into a stable, intensely colored chromophore directly within BPEEL extracts using a microplate-based, microliter-scale assay that is fully compatible with complex plant matrices and explicitly accounts for matrix effects. From an analytical chemistry perspective, this method advances the field by providing a rapid, low-cost, and solvent-saving alternative to conventional chromatographic techniques for the determination of DA in food waste. By significantly reducing the analysis time and solvent consumption and avoiding the use of derivatization agents or advanced materials, the assay represents a promising tool for sustainable food analysis and waste valorization. Overall, these features make the method well-suited for the rapid, cost-effective, and environmentally conscious quantification of bioactive compounds in food and food-waste matrices.

## 2 Experimental Section

### 2.1 Chemicals and Reagents

Water, methanol (MeOH), DMSO, and formic acid (FA, ≥98%) were of HPLC grade. All of them were obtained from Merck (Merck KGaA, Darmstadt, Germany), as well as the commercial analytical standards of DA, and Mg­(OAc)_2_ and NH_4_Cl. All solutions were prepared using water obtained from the Milli-Q Water Purification System (resistivity ≥18 MΩ cm) (Germany, www.merck millipore.com).

### 2.2 Sample Preparation

The collected banana samples belong to the Cavendish cultivar. Banana samples were prepared by manually peeling the fruit (BPEEL) and slicing the pulp into pieces (BPULP). The samples were immediately subjected to a preliminary freezing step at −20 °C for 24 h. Following preliminary freezing, the samples were transferred to a laboratory freeze-dryer (−20 °C). The freeze-drying was carried out under a chamber pressure about 0.1 mbar, with the condenser temperature maintained below −55 °C for 24 h. 1 or 5 g of the resulting freeze-dried banana pulp (BPULP) or peel powder was extracted using different solvent mixtures: 100% methanol (MeOH), 90:10 or 80:20 MeOH/H_2_O, respectively. DA matrix extraction was performed by maceration at 55 °C for 60 or 15 min. BPEEL and BPULP extracts’ solutions were filtered through a 0.45 μm membrane filter (Merck KGaA, Darmstadt, Germany) and eventually further cleaned up using a solid phase extraction step, in this case SPE-C18 column (Phenomenex, Torrance, CA, USA). Finally, the purified extracts were evaporated to dryness and stored at −20 °C. All extraction procedures were performed on three independent batches (*n* = 3) of BPEEL and BPULP samples, and each extract was subsequently analyzed in triplicate.

### 2.3 Instrumental Analysis

#### 2.3.1 Spectrophotometric Analysis of DA and BPULP and BPEEL Extracts by Optical Assay

DA standard solutions (7–160 μg/mL) were prepared in 0.5 mL of DMSO. Separately, an aqueous solution containing 300 mM Mg­(OAc)_2_ and 300 mM NH_4_Cl was prepared and the pH adjusted to 9.4 using 1 N NaOH, removing Mg­(OH)_2_ precipitate by centrifugation at 10,000 *g* for 5 min. For the assay, 100 μL of the DA standard solution or the BPULP extract or BPEEL extract were mixed with 100 μL of the aqueous solution containing 300 mM Mg­(OAc)_2_ and 300 mM NH_4_Cl in a 96-well microplate. All reactions were performed in triplicate. Control wells containing only the solvents (1:1 DMSO in the presence of 150 mM Mg­(OAc)_2_/150 mM NH_4_Cl) pH 9.4) were included as background absorbance. The absorbance peak of MN at 595 nm was monitored at 25 °C under kinetic conditions for up to 60 min. Full UV–vis spectra were acquired from 250 to 650 nm. Based on the observed kinetics, a 25 min incubation was selected as the end point for subsequent measurements. Optical measurements were performed using a SPECTROstar Nano microplate reader (BMG Labtech, GmbH, Ortenberg, Germany).

The formation of MN from DA, evidenced by a blue color development, was evaluated and optimized assessing matrix effects. For quantification of DA in BPEEL extracts, the standard addition method was applied. Each well was loaded with 50 μL of BPEEL (prepared at 16000 μg/mL in DMSO), 50 μL of DA standard solution in DMSO (11 different concentration 28–640 μg/mL), and 100 μL of the aqueous solution containing 300 mM Mg­(OAc)_2_ and 300 mM NH_4_Cl (pH 9.4), for a total reaction volume of 200 μL. Under these conditions, the BPEEL and DA additions were diluted 1:4, resulting in a final concentration of 4000 μg/mL and 7–160 μg/mL in the well.

All experiments were performed on independent extraction batches (*n* = 3) and analyzed in triplicate. Analytical performance was evaluated in terms of linearity, precision (% RSD), accuracy, recovery, and LOD and LOQ, in accordance with accepted analytical method validation criteria.

#### 2.3.2 UPLC-DAD Analysis

##### 2.3.2.1 DA Quantification Using the External Standard Method

The DA external standard method was adopted for the first preliminary evaluation of DA content in all the BPEEL and BULP extracts reported in Table 1. The UPLC-DAD system used for the optimized method was Shimadzu UPLC Nexera series constituted by a binary pump (LC-30AD), autosampler (SIL-30AC) a degassing unit (DGU-20A5), and a diode array detector (SPD-M20A) (Shimadzu, OR, USA). For data processing, Shimadzu LabSolutions software LC-GC version 5.111 (Shimadzu, OR, USA) was used. UPLC was performed using a Kinetex Polar C18 column (2.6 μm; 150 × 4.6 mm, Phenomenex, Torrance, CA, USA), under isocratic elution conditions. The mobile phase consisted of Line A: phosphate-buffered saline (PBS 10 mM) buffer at pH 2.5, and Line B: methanol (MeOH). The elution was carried out at 100% B for 10 min under isocratic conditions. Following each run, a 20 min washing cycle was applied, consisting of a 10 min linear gradient from 100% A to 100% B, followed by a 10 min return gradient from 100% B to 100% A. The flow rate was set to 0.4 mL/min. Chromatographic detection was performed at 280 nm (maximum DA absorbance), with diode array detection (DAD) over the 200–450 nm range. The column and autosampler temperatures were maintained at 15 °C, and the injection volume was 10 μL. The calibration curve of standard DA (7–160 μg/mL) was obtained with standard solutions analyzed in triplicate (*n* = 3). Quantification was performed using a linear calibration curve on standard DA using the external standard method (*m* = 12824 ± 80; a = −18511 ± 4122; *R* ^2^ = 0.9998 Table 1). DA quantification was expressed as DAE (μg DAE/g dry matrix). All experiments were performed on independent extraction batches (*n* = 3) and analyzed in triplicate. The analytical performance was evaluated in terms of linearity, precision (% RSD), accuracy, recovery, and LOD and LOQ, in accordance with accepted analytical method validation criteria.

**Table 1:** Analytical Parameters of the UPLC-DAD Method Developed for DA Quantification in BPEEL Extracts

parameters	DA in buffer	^a^DA in BPEEL
linear range (μg/mL)	7–160	7–160
slope (m)	12824 ± 80	10408 ± 122
intercept (a)	–18511 ± 4122	868018 ± 7223
correlation coefficient (*R* ^2^)	0.9998	0.9997
LOD (μg/mL)	1.06 ± 0.01	1.52 ± 0.01
LOQ (μg/mL)	3.22 ± 0.02	4.61 ± 0.03
retention time (rt, min)	2.19 ± 0.22	2.08 ± 0.12
peak area _av_RSD (%, *n* = 3)	5.30	1.76
accuracy (%)	99.00	98.00

a DA spiked in BPEEL matrix extracts (10000 μg/mL).

##### 2.3.2.2 DA Quantification Using DA Standard Addition in the BPEEL Matrix Method

The standard addition method was applied for evaluating the intrinsic DA content present in the selected BPEEL matrix extract. The BPEEL extract (10000 μg/mL) was spiked with 11 increasing concentrations of DA standard solution (7–160 μg/mL). Each concentration level was analyzed in triplicate using the developed UPLC-DAD method reported in 2.3.2.1 paragraph, under the previously described chromatographic conditions. Quantification was performed using a calibration curve constructed in matrix spiked with standard DA as reported in Table 1 (*m* = 10408 ± 122; *a* = 868018 ± 7223; *R* ^2^ = 0.9997). DA quantification was expressed as DAE (μg DAE/g dry matrix).

##### 2.3.2.3 Validation of UPLC Methods

All the parameters determined in order to validate both the UPLC analysis methods are reported in Table 1. Thus, selectivity, reproducibility, linearity, limits of detection and quantification, precision accuracy, and robustness have been determined. The intraday precision of the method was estimated by repeating calibration and standard addition curves on the same day (intraday) and on separate days (interday precision) (Figure S1) assessing intermediate precision; accuracy was evaluated at 24, 36, and 53 μg/mL and reported as average % recovery of the spiked amounts (Table 1). Method robustness was evaluated considering variations in method parameters to assess the effects of changes in the eluent concentration (PBS 10 mM and PBS 20 mM) and mobile phase flow rate (Figure S2). All experiments were performed on independent extraction batches and analyzed in triplicate. Analytical performance was evaluated in terms of linearity, precision (% RSD), accuracy, recovery, and LOD and LOQ, in accordance with accepted analytical method validation criteria. The limits of detection (LOD, 3.3 × *m*/σ) and quantification (LOQ, 10 × *m*/σ) were estimated based on the error of the intercept (σ) and the slope of the calibration curve (m) for DA in buffer or spiked in BPEEL matrix extracts. Method selectivity and specificity were confirmed by chromatographic analysis (UPLC-DAD), based on comparative analysis among BPEEL and BPULP retention time and spectral agreement with the DA reference standard. The absence of any detectable signal has been shown in BPULP extracts, where dopamine is not present at measurable levels.

### 2.4 Statistical Analysis

All experiments were performed on independent extraction batches and analyzed in triplicate presented as mean ± standard deviation (SD) (*n* = 3). The analytical performance was evaluated in terms of linearity, precision (% RSD), accuracy, recovery, and LOD and LOQ, in accordance with accepted analytical method validation criteria. One-way ANOVA followed by Tukey’s HSD test was applied where appropriate to compare extraction conditions (Table 2). Method comparison between colorimetric and UPLC-DAD data (Table 3) was evaluated using paired statistical analysis, as described in the results section. Statistical analysis was performed using GraphPad Prism software ver. 10.2.

**Table 2:** DA Extraction from BPEEL Dry Matrices at 55 °C. Values are Expressed as Mean ± Standard Deviation (*n* = 3)

solvent	time (min)	^a^treatment	dry BPEEL (g)	^b^DAE (μg/g)
MeOH	60	F/E	1	900 ± 60^c^
MeOH	15	F/E	1	1100 ± 100^c^
MeOH/H_2_O (8:2)	15	F/E	1	1650 ± 60^d^
MeOH/H_2_O (9:1)	15	F/E	1	1900 ± 20^d^
MeOH/H_2_O (9:1)	15	SPE/F/E	5	3900 ± 130^e^

a Filtration (F), evaporation (E), solid-phase extraction (SPE).

b μg of DA per g of dry matrix. Different superscript letters (c,d,e) within the DAE column indicate significant differences according to one-way ANOVA followed by Tukey’s HSD posthoc test (*p* < 0.05).

**Table 3:** Quantification of DA in BPEEL^b^

method	^a^DA (μg/g)	[ref]
colorimetry	5000 ± 200	this work
UPLC-DAD	5700 ± 300	this work
HPLC-ECP	800–5600	^9^
HPLC-DAD	950–3810	^29^
LC–MS	qualitative	^30^

a μg of DA per g of dry matrix.

b Values are expressed as mean ± SD (*n* = 3).

## 3 Results and Discussion

In this study, we report the development of a rapid, simple, and cost-effective method for the detection of DA in food and food waste matrices, using banana as a case study. DA is a key bioactive compound in bananas, and its distribution varies markedly between different parts of the peel and pulp, with particularly high concentrations in the peel (∼700 μg/g) and moderate levels in the pulp (∼8 μg/g).^9^ Beyond DA, the bioactive compounds extracted from dried BPEELs, especially phenolics and flavonoids, are of growing interest for their antioxidant properties, which can be harnessed in the development of functional foods and nutraceuticals aimed at improving human health.^17,18^ Given the large global consumption of bananas, this work highlights the potential of DA quantification as a valuable contribution to agricultural food waste valorization.

To this end, we first set up a rapid UPLC-DAD analysis method for the analysis of DA content (external standard method) and applied it to banana sample extracts (BPEEL and BPULP) to assess the presence of DA. Our results confirmed that DA is predominantly present in the peel, which led us to optimize the extraction procedure to maximize its recovery. The extract with the highest DA content was selected for the adaptation of a colorimetric assay based on the formation of MN, a purple-blue chromophore in a DMSO/H_2_O (1:1, v/v) mixture, in the presence of 150 mM Mg­(OAc)_2_ and 150 mM NH_4_Cl at pH 9.4. This assay, previously developed for pharmaceutical applications,^16,19^ was recalibrated and applied to the banana matrix, including the evaluation of matrix effects and calibration curve generation.

Finally, we compared the colorimetric method with an UPLC method specifically adapted to account for DA for matrix effects—moving beyond external standard calibration—to ensure precise quantification and reliable comparison. Results from chromatographic and colorimetric approaches are here compared, and the main analytical parameters of both methods discussed.

### 3.1 Development of the UPLC-DAD Method for DA Content Evaluation in BPEEL and BPULP

Banana samples (BPEEL or BPULP as freeze-dried powders prepared from fresh bananas, as described in the Materials and Methods section) were used as the starting material (1 or 5 g) for DA extraction by maceration in MeOH or a hydroalcoholic solution.

For DA quantification in all the resulting extracts, a new and rapid UPLC-DAD method was developed and validated based on external standard calibration. Optimal chromatographic performance was achieved under isocratic elution using a DMSO/H_2_O (1:1, v/v) sample diluent and a 100% PBS 10 mM mobile phase at pH 2.5, which suppressed DA ionization and provided sharp, symmetric peaks with a retention time of 2.19 ± 0.20 min (Table 1). The method showed good repeatability (RSD = 5.3%, *n* = 3) and excellent linearity over the concentration range of 7–160 μg/mL (*R* ^2^ = 0.9998) (Table 1, Figure S1A). The LOD and LOQ were 1.06 ± 0.01 μg/mL and 3.22 ± 0.02 μg/mL, respectively. Precision was confirmed through intra- and interday calibration curves, with strong superimposition (Figure S1B). Robustness tests involving slight variations in the flow rate and PBS concentration showed no significant effect on quantification (Figure S2). These results confirm the method’s suitability for the sensitive and accurate quantification of DA in complex plant-derived matrices.

#### 3.1.1 Optimization of DA Extraction in BPEEL and BPULP

To optimize DA extraction condition from banana tissues, different methanol/water systems (MeOH, MeOH/H_2_O 9:1, and MeOH/H_2_O 8:2) and extraction times (15 and 60 min) at 55 °C were tested on BPULP and BPEEL (Table 2). Extract treatment includes filtration step (F) or purification using solid-phase extraction (SPE), followed by solvent evaporation. This validated method was then applied to assess the effectiveness of the various extraction conditions from BPEEL and BPULP, enabling direct comparison of DA recovery. DA was not detectable in BPULP extracted with MeOH at 55 °C for 60 min, likely due to low DA concentration in pulp (∼8 μg/g), as reported in literature,^7,9^ below the LOD of 1.06 μg/mL. In contrast, the DA concentration in BPEEL, extracted under the same conditions, was 900 ± 60 μg/g (Table 2), consistent with previously reported values for this cultivar (∼700 μg/g),^9^ as further supported by the chromatographic profiles at 280 nm in PBS pH 2.5 for BPEEL, BPULP, and DA standards (Figure 1). DA standard (purple in Figure 1) and BPEEL (blue in Figure 1) share the main peak about 2 min that is missing in BPULP chromatogram (green in Figure 1), appearing flat. The broad peaks observed in the chromatograms of banana extracts reflect the partial overlap of multiple components naturally present in the matrix. Among these, DA represents the major contributor, and its elution corresponds to the main peak in the chromatographic profile. Nevertheless, the quantitation was based on the dominant DA signal, whose retention time and spectral characteristics were consistent with the pure standard, ensuring reliable quantification. These results confirm the reliability of both the extraction and analytical protocols. Accordingly, subsequent discussions will focus exclusively on BPEEL extracts. Reducing the extraction time to 15 min, under the same solvent (MeOH) resulted in slightly higher DAE content in the peel (1100 ± 100 μg/g) (Table 2). This supports the hypothesis that DA undergoes partial thermal degradation during prolonged extraction at 55 °C, even in 100% alcoholic media, and that shorter extraction times help preserve analyte stability. Extraction temperature and time were systematically evaluated to account for the known oxidation sensitivity of DA. Short extraction times (15 min at 55 °C) were selected as a compromise between extraction efficiency and analyte stability as longer extraction times resulted in lower DA recovery, likely due to partial thermal or oxidative degradation.

**Figure 1:**
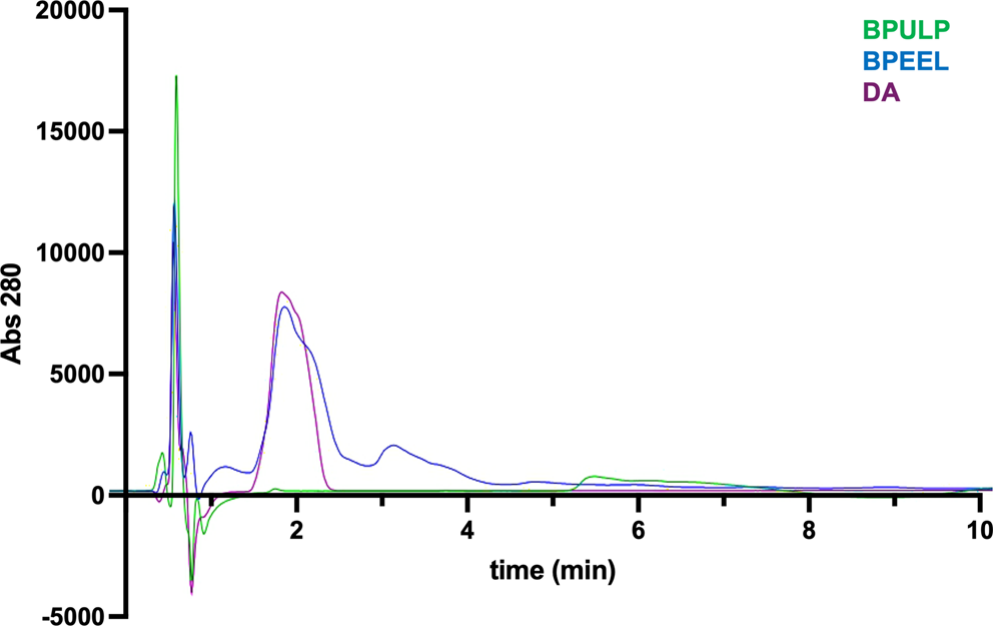
Chromatograms from UPLC-DAD analysis (280 nm) of DA in standard solution (purple), BPEEL (blue), BPULP (green) extracts evaluated by the external standard method (mobile phase 100% PBS buffer 10 mM pH 2.5). Peak broadening reflects matrix complexity. See the materials and methods (2.3.2.1 section) for further details.

To further enhance the extraction efficiency, hydroalcoholic solvent mixtures were explored. Using MeOH/H_2_O (8:2, v/v) increased DAE yield to 1650 ± 60 μg/g, while a 9:1 mixture significantly improved recovery to 1900 ± 20 μg/g (Table 2). This trend suggests that a small proportion of water enhances DA solubility by better matching the solvent’s polarity to that of the analyte, without significantly promoting oxidative degradation, in agreement with literature indicating that moderately polar environments favor catecholamine extraction while minimizing oxidative stress.^20^ Finally, process scale-up using 5 g of dried BPEEL and SPE cleanup, instead of the simple filtration in MeOH/H_2_O (9:1), yielded the highest DAE concentration observed (3900 ± 130 μg/g). This indicates that SPE significantly improves analyte recovery enhancing purification. Overall, DA extraction from BPEEL is strongly influenced by solvent polarity, extraction time, and cleanup strategy. The optimal conditions—short extraction time, hydroalcoholic solvent (MeOH/H_2_O 9:1), and SPE cleanup—provided the best extract for subsequent colorimetric analysis, highlighting the importance of balancing extraction efficiency with analyte stability when targeting labile bioactive molecules such as DA in plant-derived matrices.

### 3.2 Colorimetric Assay Development

After the optimization of extraction conditions and preliminary quantification of DAE by UPLC-DAD, the focus shifted to the development of a complementary colorimetric assay for DA detection in BPEEL extracts. This assay is based on the oxidative conversion of DA to MN, a stable chromophore that produces a visible purple/blue color and can be quantitatively detected at 595 nm.^14^ In this assay, the formation of MN from DA in DMSO/H_2_O 1:1 (v/v) in the presence of 150 mM Mg­(OAc)_2_ and 150 mM NH_4_Cl at pH 9.4 was readily detectable by the development of an intense purple/blue coloration. MN formation is the result of DA oxidation under mildly alkaline conditions, where DA auto-oxidizes to form colored quinone structures.^21,22^ The use of MN as a visible chromophore provides a direct and specific readout for DA detection within the assay. Although this strategy was originally developed for pharmaceutical applications,^16,19^ in this study it has been innovatively adapted and validated for the analysis of banana extracts, serving as a visual and quantifiable marker of DA content in matrices relevant to food waste valorization. This adaptation represents a significant advancement, extending the method’s applicability beyond its original scope and demonstrating its potential as a robust, accessible, and cross-disciplinary analytical tool. Within the food waste valorization framework, the approach enables reliable detection and quantitation of valuable compounds in food waste, thereby supporting their efficient valorization into nutraceuticals, functional foods, and bioproducts.

To ensure optimal performance under the experimental conditions used in this study, the assay parameters were first assessed. The kinetics of MN formation were evaluated for DA (50 μg/mL) in 150 mM Mg­(OAc)_2_, 150 mM NH_4_Cl at pH 9.4. The kinetic profile showed a rapid increase of absorbance at 595 nm within the first 25 min, reaching 80% of the absorbance achieved after 60 min (Figure 2A). The stability of the absorbance over long time indicates the absence of interfering side reactions or degradation, supporting the assay’s specificity and reliability. Based on these kinetics, a 25 min incubation time was selected as optimal for subsequent analyses, offering a balance between rapid signal development and analytical reproducibility to ensure consistent MN formation and improved comparability across samples. As the method is designed as a rapid end point colorimetric assay, extended stability studies over prolonged periods were not considered necessary for its intended application.

**Figure 2:**
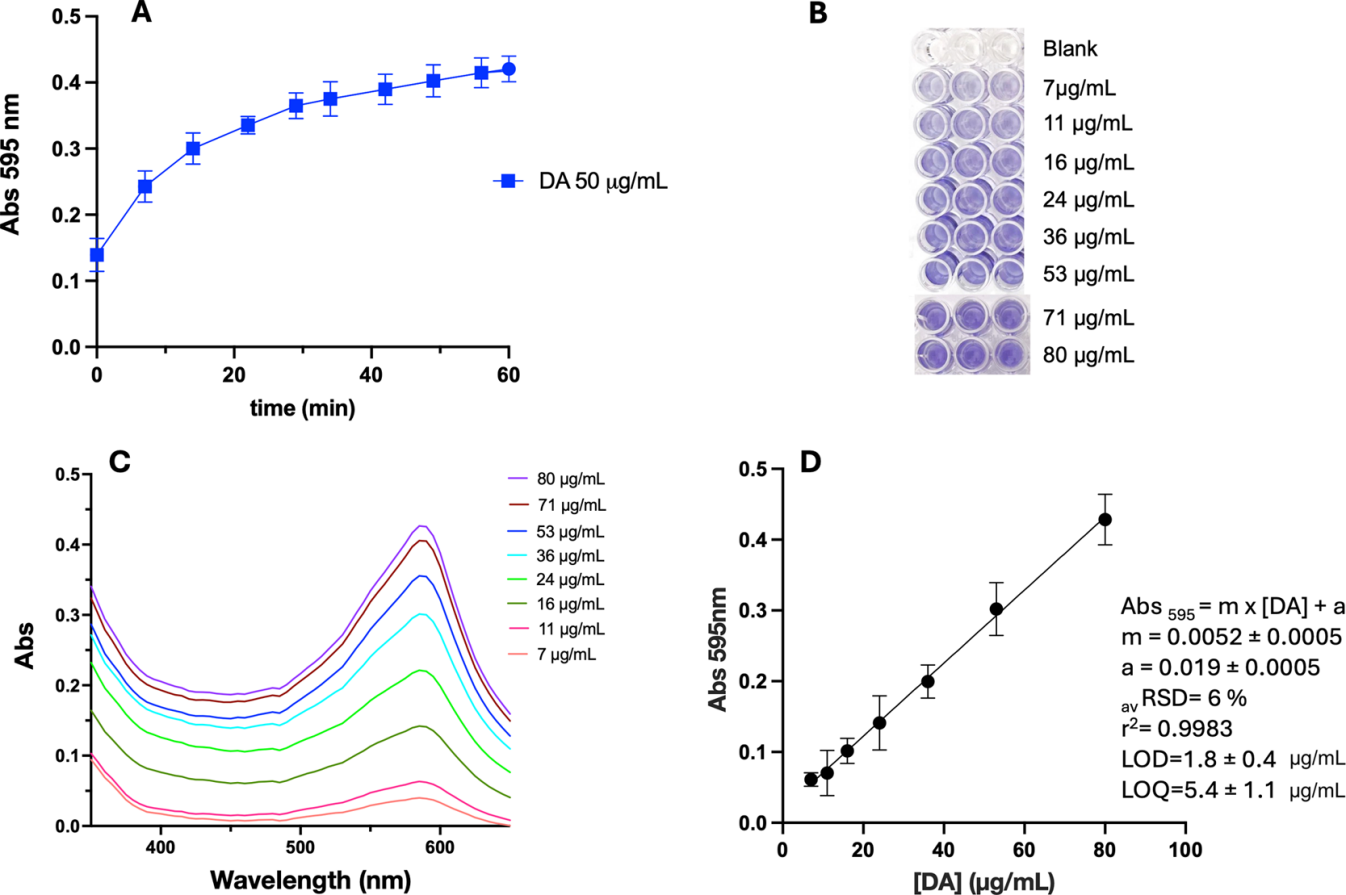
Time-dependent oxidation of DA and MN chromophore formation with calibration and spectral analysis under assay conditions. Time-dependent oxidation of DA 50 μg/mL in DMSO/H_2_O 1:1 (v/v) in the presence of 150 mM Mg­(OAc)_2_, 150 mM NH_4_Cl at pH 9.4 and 25 °C between 0 and 60 min (A). Dose-dependent blue/purple color development due to MN formation in 96 wells with DA calibration curve under assay conditions (B). UV–visible spectra (C) and calibration curve (D) for DA (7–160 μg/mL) in DMSO/H_2_O 1:1 (v/v) in the presence of 150 mM Mg­(OAc)_2_, 150 mM NH_4_Cl at pH 9.4 and 25 °C after 25 min incubation.

A calibration curve for DA (7–160 μg/mL) was obtained measuring absorbance at 595 nm at 25 °C after 25 min of incubation in DMSO/H_2_O 1:1 (v/v) containing 150 mM Mg­(OAc)_2_, 150 mM NH_4_Cl at pH 9.4 (Figure 2). A distinct purple color rose up due to MN formation at concentrations above 11 μg/mL (Figure 2B,C). The linear regression equation for absorbance data from visible spectra in Figure 2D was Abs_595 nm_ = 0.0052×[DA] + 0.019, with an excellent correlation coefficient (*R* ^2^ = 0.9983), confirming strong linearity across the tested range (Figure 2D). The slope (*m* = 0.0052) reflects satisfactory sensitivity, and the intercept (*a* = 0.019) indicates minimal background signal under the assay conditions. Analytical performance was further assessed in terms of precision and sensitivity. The average relative standard deviation (_av_RSD) across replicates was 6%, demonstrating good intra-assay reproducibility, particularly for a microplate-based colorimetric method. The LOD and LOQ were calculated as 1.8 ± 0.4 μg/mL and 5.4 ± 1.1 μg/mL, respectively, confirming the high sensitivity of the assay without requiring derivatization or complex sample enrichment.

#### 3.2.1 Detection of DA in the BPEEL Extract by Colorimetric Assay

To verify the presence of DA in BPEEL extracts, the developed colorimetric assay conditions (150 mM Mg­(OAc)_2_, 150 mM NH_4_Cl, pH 9.4, 25 °C, 25 min incubation) were applied. The BPEEL extract with the highest DAE concentration (3900 ± 130 μg/g; Table 2) was analyzed alongside the BPULP extract and a DA standard, as shown in Figure 3. The BPEEL extract (10000 μg/mL) developed a purple/blue chromophore, exhibiting a distinct absorbance peak at 595 nm comparable to that of DA standard solution (200 μg/mL). This result indicates the presence of DA in the peel undergoing similar oxidative conversion to MN, under the assay conditions. In contrast, the BPULP extract (10000 μg/mL) showed no absorbance band at 595 nm, in agreement with the UPLC-DAD analysis (Figure 1). This observation confirms the selectivity of the assay, suggesting that DAE concentrations in BPULP are below the detection limit and that other coextracted polyphenolic compounds do not significantly interfere with the assay response. These findings confirm that DA is predominantly localized in banana peel, where it may contribute to defense mechanisms, pigmentation, and postharvest browning.^8,9^

**Figure 3:**
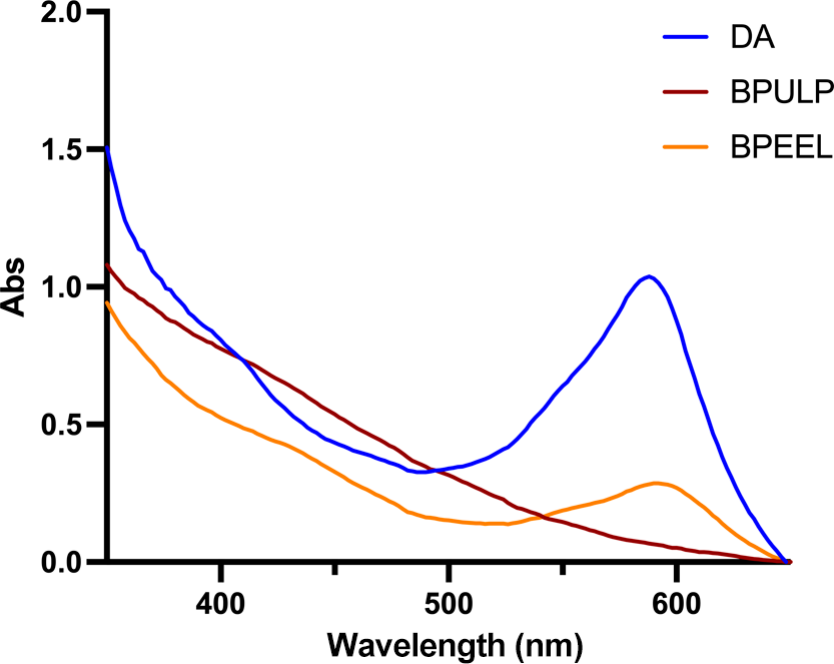
Visible spectra superposition for MN formation from DA (200 μg/mL), BPULP (10000 μg/mL), and BPEEL (10000 μg/mL). Spectra were acquired in DMSO/H_2_O (1:1, v/v) containing 150 mM Mg­(OAc)_2_ and 150 mM NH_4_Cl at pH 9.4 after 25 min incubation.

Following the initial screening of both BPULP and BPEEL, the analysis was focused on demonstrating DA-accurate quantification on BPEEL by the colorimetric assay. Thus, the method was applied for the quantitative determination of DA in BPEEL via the standard addition method. BPEEL is known to contain several oxidizable phenolic compounds; however, DA is the only naturally occurring catecholamine present at milligram/g levels in this matrix. Under the specific alkaline Mg^2+^/NH_4_^+^ conditions employed, DA is the dominant species that undergoes oxidative conversion to MN. This interpretation is supported by the absence of a detectable signal at 595 nm in the BPULP extract, which contains phenolics but negligible DA levels, and by the close agreement between DA quantification obtained by the colorimetric assay and by matrix-matched UPLC-DAD analysis (Figures 1 and 3). While minor contributions from other oxidizable catechol-type compounds cannot be completely excluded, they do not significantly affect DA quantification in BPEEL under validated experimental conditions.

#### 3.2.2 Quantification of DA in the BPEEL Extract by Colorimetric Assay

The quantification of DA in BPEEL extracts was carried out using the standard addition method, thereby demonstrating the applicability of the developed colorimetric assay to food waste (FW) matrices. Eventually, the results have been compared with chromatographic analysis (see 3.3 paragraph), performed on the same samples. Aliquots of the BPEEL extract (4000 μg/mL) were spiked with DA standard solutions (7–160 μg/mL) in DMSO/H_2_O (1:1, v/v) containing 150 mM Mg­(OAc)_2_ and 150 mM NH_4_Cl at pH 9.4, resulting in the characteristic color development shown in Figure 4A. After 25 min of incubation, absorbance spectra were recorded (Figure 4B), and the peak at 595 nm—corresponding to MN formation—was plotted against the spiked DA concentrations (Figure 4C). The curve obtained showed a linear dynamic range–50 μg/mL DA and was fitted by a linear regression equation described by Abs_595 nm_ = 0.01×[DA] + 0.20, with a correlation coefficient *R* ^2^ = 0.9974, indicating excellent linearity in the tested concentration range, even in the presence of the complex sample. The slope (*m* = 0.01) was slightly higher than that observed under standard conditions, possibly reflecting a mild enhancement of chromophore formation in the presence of BPEEL constituents. The method displayed high repeatability in the matrix, with an average relative standard deviation (_av_RSD) of 1.94%. Based on the extrapolated data, the DA content in the analyzed BPEEL extract, expressed as DAE, was estimated to be 5000 ± 200 μg DAE/g dry BPEEL. These findings validate the assay’s applicability to real matrices and its potential for use in food waste valorization or natural product profiling workflows, particularly in view of its operational simplicity, minimal solvent use, and compatibility with microplate formats.

**Figure 4:**
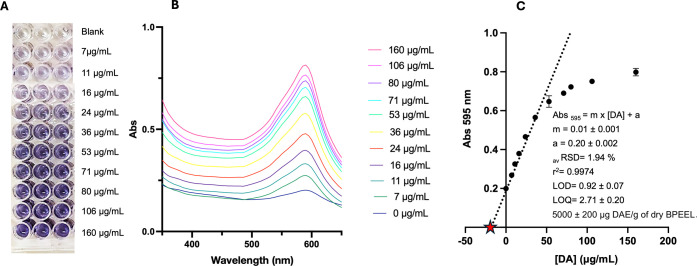
Dose-dependent blue/purple color development due to MN formation in 96 wells with BPeel extract spiked with DA in standard addition calibration curve under assay conditions (A). UV–visible spectra (B) and standard addition curve (C) for DA (7–160 μg/mL) in the presence of BPEEL extract 4000 μg/mL in DMSO/H_2_O 1:1 (v/v), 150 mM Mg­(OAc)_2_, 150 mM NH_4_Cl at pH 9.4 and 25 °C after 25 min of incubation.

### 3.3 Colorimetric Assay Validation on the BPEEL Matrix by DA Standard Addition using the UPLC-DAD Method

Finally, both the colorimetric assay and chromatographic analysis (UPLC-DAD) were applied to spiked BPEEL samples to enable the accurate quantification of DA in the extract. While the external standard method provided an initial estimate of the DA content, potential matrix effects in BPEEL could compromise measurement accuracy. To address this, a matrix-matched calibration was performed using the standard addition method, ensuring reliable DA determination within the real sample matrix.

Thus, the BPEEL sample (10000 μg/mL) was spiked with DA standard (7–160 μg/mL). As reported in Table 1 and Figure 5, the method exhibited excellent linearity over the entire DA concentration range, with excellent linearity with *R* ^2^ values of 0.9997. The method’s detection and quantification limits (LOD = 1.52 ± 0.01 μg/mL; LOQ = 4.61 ± 0.03 μg/mL) in matrix remain suitable for the expected DA levels in banana waste. Precision was confirmed with _av_RSD­(%) values below 1.76% in matrix (Table 1), indicating high repeatability. The accuracy, evaluated through standard addition, yielded excellent recovery values (98.5–99.9%) across spiked concentrations (Table 1 and Figure S1), further validating the quantification strategy despite matrix complexity. The calibration curves intra- and interday, with strong superimposition confirm excellent reproducibility (Figure 5B). The method allowed a quantification of 5700 ± 300 μg of DAE per g of BPEEL. Although it is well established in the literature that BPEEL contains high levels of polyphenols, particularly phenolic acids and flavonoids bearing catechol groups, DA represents the main naturally occurring catecholamine that could give the peculiar oxidative conversion to MN in BPEEL extracts.^23–25^ Notably, the quantification of DA via UPLC using a matrix-matched calibration resulted in higher measured concentrations compared to the external standard method in Table 1, likely due to matrix effect. In the case of BPEEL extracts, coextracted components may interfere with the chromatographic or detection process—either by suppressing the analyte signal or by altering its extraction or chromatographic behavior—leading to an underestimation of the actual concentration when using a calibration performed in pure solvent. By replicating the matrix environment in the calibration standards, the matrix-matched approach compensates for these effects, allowing for a more accurate and representative quantification. This result further supports the need to account for matrix effects in quantitative analyses of complex food substrates, particularly when applying spectroscopic or chromatographic methods to real-world samples.

**Figure 5:**
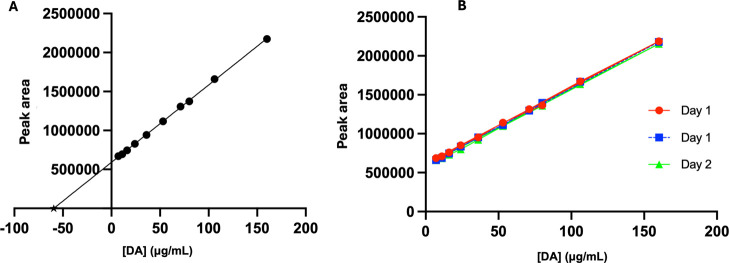
UPLC-DAD calibration curves for DA (7–160 μg/mL) standard solutions (A) or spiked in the BPEEL extract (10000 μg/mL) (B), including intraday analysis of the DA calibration curve.

The quantification of DA in BPEEL using both colorimetric assay and UPLC techniques and data from literature are reported in Table 3. The DA content determined by colorimetric assay was 5000 ± 200 μg DAE/g dry BPEEL, whereas UPLC analysis yielded a slightly higher value of 5700 ± 300 μg/g, confirming the accuracy and reproducibility of our colorimetric assay based on MN formation, and supports its use as a viable, low-cost alternative to chromatographic instrumentation for preliminary screening. A paired *t*-test comparing DA detection in colorimetry and UPLC-DAD data (*n* = 3) reported in this work showed no statistically significant difference between the two methods. When compared to literature values reporting DA concentrations in BPEEL ranging from 0.8 to 5.6 μg/mg, the values obtained in this study fall at the upper end of that range. This result may reflect either a particularly DA-rich sample or enhanced extraction efficiency achieved under the optimized MeOH/H_2_O (90:10) conditions (Table 2). The application of elevated temperature (55 °C) and solid-phase extraction (SPE) likely contributed to improved recovery, aligning with recent findings that highlight the critical role of solvent polarity and purification steps in maximizing catecholamine stability and extraction yield.

From an economic perspective, the comparison reported in Table 3 highlights that the proposed colorimetric assay requires simpler instrumentation, lower reagent, and solvent consumption, and significantly shorter analysis times compared to the UPLC-DAD method. A limitation of the present study is that, as a colorimetric approach, the method is primarily intended for rapid screening rather than detailed molecular characterization. While it provides a simple, fast, and cost-effective analytical tool for the quantification of dopamine equivalents in complex matrices, it does not offer the level of molecular specificity achievable with advanced techniques such as LC–MS/MS. Therefore, its application is best suited for preliminary analysis and high-throughput screening. In addition, unlike several previously reported colorimetric approaches based on enzymes or nanomaterials,^11,26–28^ the present assay relies on inexpensive reagents and straightforward procedures, making it a cost-effective option for routine screening and preliminary quantification of DA in food and food-waste matrices.

## 4 Conclusions

Overall, in parallel with UPLC, we developed a μL-scale colorimetric assay employing minimal solvent volumes (DMSO/H_2_O, Mg^2+^/NH_4_^+^ buffer) and a short extraction time (15 min at 55 °C), thereby significantly reducing solvent consumption and minimizing laboratory waste. The method proved accurate, reproducible, and directly applicable to real matrices, establishing it as a cost-effective and fast alternative to chromatographic instrumentation. Importantly, the high DA levels detected in BPEEL extracts underscore their potential as a valuable source of natural antioxidants, directly linking analytical innovation to food waste valorization.

The simplicity and portability of the assay make it particularly suitable for rapid onsite screening in food processing facilities and waste management plants, enabling fast decision-making on food waste valorization and bioactive recovery. Beyond DA, the bioactive compounds extracted from dried BPEELs, especially phenolics and flavonoids, are of growing interest for their antioxidant properties, which can be harnessed in the development of functional foods and nutraceuticals aimed at improving human health.

From an environmental perspective, the utilization of BPEELs, typically discarded as waste, highlights the importance of sustainable practices in food-processing. By transforming waste into valuable resources, this approach contributes to the circular economy, reducing the environmental burden while generating economic and nutritional value.

The developed colorimetric assay thus provides an essential analytical tool to support this transition, enabling the food industry to efficiently screen the bioactive potential of banana peels and other complex matrixes. This strategy fosters a comprehensive approach to waste management and resource recovery, fully aligned with the principles of sustainable chemistry, which emphasize environmentally friendly extraction methods that preserve bioactive integrity while minimizing the energy consumption.

## Supplementary Material



## Notes

UPLC-DAD calibration curve for DA (7–160 μg/mL) at 280 nm; interday/intraday analysis of the DA calibration curve in DMSO/H_2_O 1:1; and effect of the flow rate on the retention time of the DA peak (mobile phase 100% PBS buffer 10 mM pH = 2.5) (PDF)
