# Supplementary material for: Colorimetric Quantification of Dopamine as a Bioactive Compound in Banana Peel Extracts: A Sustainable Approach to Food Waste Valorization
Source: ACS Omega. 2026 Jun 15;11(25):37988–96. doi: 10.1021/acsomega.6c03329 (PMC13325377; doi:10.1021/acsomega.6c03329)

Supplemetary Material

Colorimetric Quantification of Dopamine as a Bioactive Compound in Banana Peel: A Sustainable Approach to Food Waste Valorization

Doretta Cuffaro*[a], Enrico Crispino [a], Elisa Nuti [a], Vincenzo Calderone [a], Pasquale Palladino [b], M. Minunni[a]

[a]Department of Pharmacy, University of Pisa, Via Bonanno 6, 56126 Pisa, Italy;
[b]Department of Chemistry 'Ugo Schiff', University of Florence, Via della Lastruccia, 3, 50019 Sesto Fiorentino (FI), Italy.
Corresponding authors: doretta.cuffaro@unipi.it;

Fig. S1. UPLC-DAD calibration curve for DA (7-160 µg/mL) at 280 nm (A). Interday/Intraday analysis of DA calibration curve in DMSO:H_2O 1:1 (B).

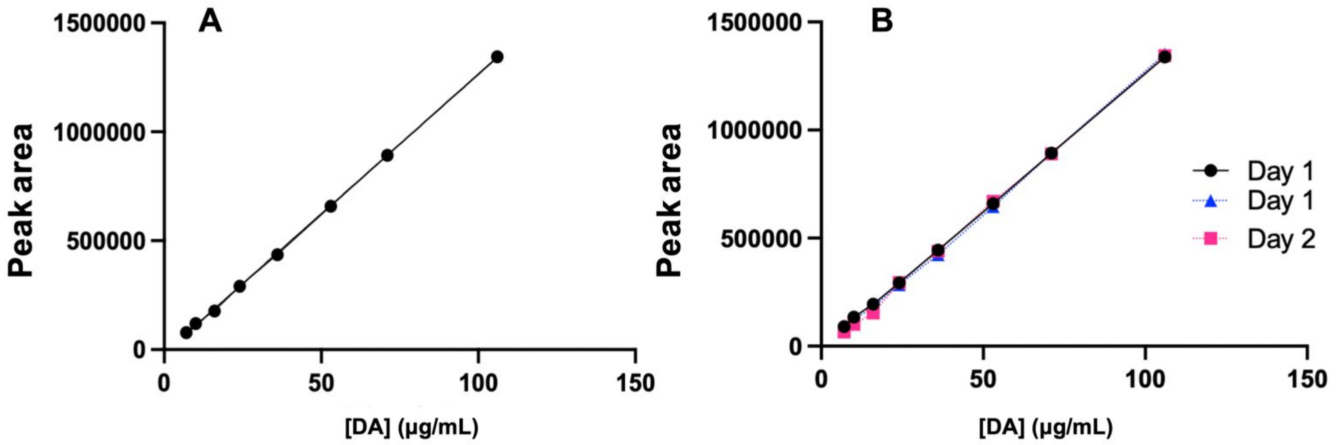

Fig. S2. Effect of flow rate on the retention time of the DA peak (mobile phase 100% PBS buffer 10 mM pH=2.5)

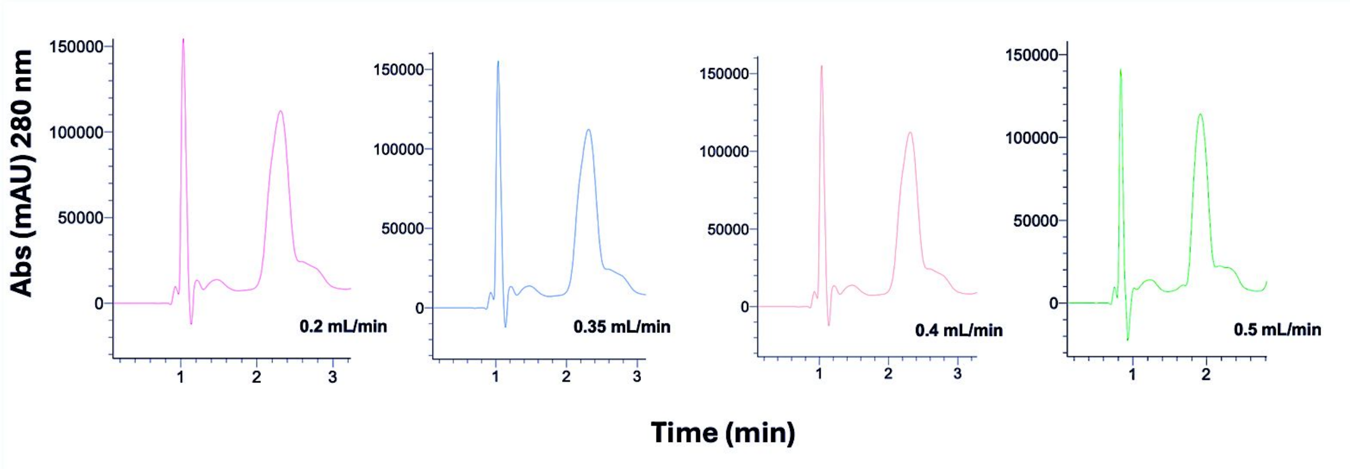

Supplement: Supplementary file 1 [file ao6c03329_si_001.pdf]
